# Surname order and revaccination intentions during the COVID-19 pandemic

**DOI:** 10.1038/s41598-024-55543-2

**Published:** 2024-02-27

**Authors:** Eiji Yamamura, Yoshiro Tsutsui, Fumio Ohtake

**Affiliations:** 1 Department of Economics, Seinan Gakuin University, Fukuoka, Japan; 2 Faculty of Social Relations, Kyoto Bunkyo University, Kyoto, Japan; 3 Center for Infectious Disease Education and Research (CiDER), Osaka University, Osaka, Japan

**Keywords:** Surname, Natural experiment, Name order, Hidden curriculum, Mixed gender list, Non-cognitive skill, Revaccination, COVID-19, Health care, Health care economics, Public health

## Abstract

Teachers in Japanese schools employ alphabetical surname lists that call students sooner, with surnames appearing early on these lists. We conducted Internet surveys nearly every month from March 2020 to September 2022 with the same participants, wherein we asked participants where the alphabetical columns of their childhood and adult surnames were located. We aimed to identify how surname order is important for the formation of noncognitive skills. During the data collection period, the COVID-19 vaccines became available; Japanese people could receive their third dose starting in December 2021. The 19th wave of the survey was conducted in January 2022. Therefore, to examine how a surname’s alphabetical order could influence intention to revaccinate, we used a subsample of data from December 2021 to September 2022. The major findings were as follows. Women with early surnames had an approximately 4% stronger likelihood of having such intentions than men with early surnames. Early name order was more strongly correlated with revaccination intention among women than among men. The surname effect for women was larger when a mixed-gender list was used compared with when it was not used. This effect was only observed for childhood surnames and not for adult surnames.

## Introduction

Alphabetical surname lists, often unintentionally employed by teachers in Japan owing to their convenience, can be viewed as a form of “hidden curricula”^1–5^. Students with surnames appearing early on these lists tend to be called upon sooner in various school situations, enabling them to learn from new experiences earlier. Conversely, those with later surnames often feel marginalized as they frequently encounter new settings after their peers, thereby impacting their future life outcomes^6^. Moreover, traditionally, male students are listed before females, irrespective of alphabetical order, with same-gender students ranked by surname. This order mirrored the male-dominant societal norm, although a shift toward mixed-gender lists has occurred since the 1980s^1–5^.

Childhood education significantly shapes precautionary and preventive behaviors against unforeseen disasters. Studies have shown that hygiene education in schools, a kind of “hidden curriculum,” cultivates non-cognitive skills related to worldviews and preferences, leading to habits such as regular hand-washing and mask-wearing during pandemics like COVID-19^7^.

In the COVID-19 era, vaccinations may affect the motivation for preventive behaviors, altering individuals’ post-vaccination actions^8–16^. However, motivation might differ between initial vaccination and revaccination, an area often overlooked by researchers focusing on attitudes towards vaccination^16–36^ which are crucial for sustaining the protective effects of vaccination, especially as the efficacy of initial shots decreases over time.

The rate of completing the first and second shots was approximately 78%^37^. However, the preventive effects of the first and second shots decreased over time. Furthermore, the third shot was effective in preventing COVID-19, particularly the newly emerged omicron variant^38–41^. Based on this evidence, the Ministry of Health, Leisure, and Welfare has publicly urged people to revaccinate^42^. However, the rates for third and fourth shots were approximately 68% and 46%, respectively^37^. The rates for the third and fourth shots were distinctly lower than those for the first and second shots. Revaccination may incur psychological costs, even for those who complete their first and second shots. Despite the importance of revaccination, many studies have considered attitudes towards vaccination, and little is known about this issue. This study examines how learning experiences in schools promote revaccination.

The hypothesis is that individuals with early-listed surnames may be more open to revaccination because of the reduced psychological costs associated with novel situations, a benefit reaped from early school experiences. We also anticipate that the “surname effect” would be stronger for males from the generation that did not experience mixed-gender lists, with male students invariably listed before female students. The adoption of mixed-gender lists is predicted to enhance female students' learning opportunities^1–5^. However, to date, no quantitative research has examined the impact of adopting mixed-gender lists, an issue we address using our independently collected panel dataset.

Our findings reveal that (1) individuals with earlier childhood surnames exhibit a stronger intention to revaccinate; (2) this correlation is larger for females if a mixed-gender list is used during their schooling; and (3) there is no correlation between adulthood surname and the intention to revaccinate. By exploring the enduring effects of implementing mixed-gender lists in schools, this study contributes to educational research, particularly in the context of unpredictable events such as the COVID-19 pandemic.

## Research background

### The alphabetical list in school

The Japanese alphabetical order is structured around a 50-character "Kana" syllabary, classified into ten columns with five characters (The first column consisted of 5 vowel letters, which ordered as “A,” “I,” “U,” “E,” and “O.” This is called as “A” column. Next to “A” column, “Ka” column comes, containing orderly “Ka,” “Ki,” “Ku,” “Ke” and then lastly “Ko.” In this way, 10 columns are ordered.) each. In Japanese primary schools, students are listed in alphabetical order according to their surnames. There are no established rules for calling students into Japanese schools, leaving them largely to teacher discretion. However, teachers often rely unconsciously on alphabetical lists to call students during class and non-academic events^1–5^. Consequently, students with early-listed surnames tend to have more frequent experiences throughout their school lives. Although the mother’s name can be inherited, the child's surname is generally chosen from that of the father. Regardless, students and parents cannot select an early surname from names other than those of their parents, indicating that the surname is given exogenously.

Japan, a male-dominated society, has seen discriminatory practices against females in workplaces, households, and various settings. For instance, the percentage of female students attending higher-level schools was lower than that of male students, with university entrance rates of 55.7% for males and 47.4% for females in 2017, albeit with the gender gap gradually decreasing over time^43^. A possible reason for this gender gap could be the disadvantages experienced by female students due to the traditional listing method. Traditionally, male students are listed first by surname, followed by female students. Therefore, all female students were listed after all male students, reflecting the traditional norms of a male-dominated society.

Thus, female students are less likely to develop cognitive and noncognitive skills because of this discriminatory listing practice^1–5^. To promote gender equality, a shift to mixed-gender lists began in the 1980s, when students were listed in alphabetical order regardless of gender. By 2017, approximately 80% of primary and junior high schools had adopted this approach^1^.

### Surname effects in related literatures

Surname use is often associated with life outcomes. The alphabetical order of surnames can influence productivity and performance outcomes, with early surnames generally improving individual performance and evaluations. Surname order and academic performance in high school were correlated^44^. Additionally, surname order was correlated with peer relationships among students in school. Earlier surnames were positively correlated with various indices such as friendship, acceptance, acquaintance, and liking^45^. This implies that early alphabetical order contributes to the formation of noncognitive and cognitive skills.

In academia, a first author with an earlier surname leads to more citations^46–48^. Earlier alphabetical order of surnames was positively correlated with better performance and evaluation, regardless of the equality of work. A similar mechanism is observed, in which the paper on the cover page is more likely to be cited and highly evaluated^49–51^. A similar phenomenon was observed in election outcomes. Candidates with earlier surnames had significant electoral advantages^52–56^.

One key factor is that people often choose from the top of a list, whether they are references in academic papers or candidates in an election. This tendency is driven by incentives to save time and effort while evaluating the quality of academic work or candidates’ campaign pledges.

Apart from the outcome of surname advantage in schools, the name is associated with firm performance^57–59^. For family firms, the presence of the founding family’s name as part of the firm's name can improve performance^58^. In addition to improved performance, firms with family names have significantly higher levels of corporate citizenship and customer representation^57^.

The surname order has a negative effect, which is regarded as a “negative externality” in the field of economics. The inequality in labor productivity caused by surname order results in wage inequality between early and late list placements. A wage gap can cause friction and conflict among workers, thereby reducing incentives. A recent study found that the alphabetical order of surnames reduces team incentives, negatively impacting output^60^.

## Data and methods

### Data collection

Our project aimed to explore how the COVID-19 pandemic influenced individuals' preventive behaviors and subjective views on prevention at the onset of the pandemic. We aimed to achieve this by conducting repeated surveys with the same individuals. Initiated in March 2020, immediately after the detection of COVID-19 in Japan, this project commissioned the research company INTAGE to collect data through online surveys. We selected it because of its extensive academic research experience.

Participants were recruited using INTAGE from individuals preregistered for the survey. The survey sample was taken from an existing sample maintained by INTAGE. The respondents were chosen randomly to meet pre-specified quotas that represented the Japanese adult population. Data on various aspects such as household income, age, sex, educational background, and area of residence were collected. The initial survey in March 2020 saw a participation rate of 54.7%. Individuals under 18 and above 78 years of age were not pre-registered as subjects by INTAGE, and hence were not included in the sample. Those over 78 years of age were considered less likely to use the internet. Therefore, the sample population was restricted to individuals aged 18–78.

We assembled the panel data as follows: we conducted internet surveys nearly every month on 26 occasions (“waves”) between March 2020 and September 2022 with the same subjects. During this period, the COVID-19 vaccines became available. These vaccines are critical in combating COVID-19 and are thus invaluable for investigating people's intentions to receive vaccines. Many studies have explored how unvaccinated people are motivated to be inoculated with COVID-19. As of March 2023, most people had received their first and second doses of the COVID-19 vaccine. It has now become crucial for vaccinated people to be revaccinated as vaccines lose effectiveness over time. Our study focused on revaccinations using a subsample of individuals who received their first and second doses of the COVID-19 vaccine. Japanese people can receive their third dose starting in December 2021. The 19th wave of the survey was conducted in January 2022. Therefore, to examine the intention to revaccinate, we use a subsample covering December 2021 (19th wave) to September 2022 (26th wave).

As provided in the Appendix of the Supplementary File (S1), the questionnaire included questions about basic demographic, social, and economic characteristics. Descriptions of the collected variables are presented in Table 1. The outcome variable was the subjective revaccination intention, posed as the question, "Will you get a vaccination shot? Choose from 5 choices. Respondents’ choices: 1 (will not get a shot) to 5 (will get a shot)." A higher value indicates a higher revaccination intention. The sample size was restricted to patients who received a second dose of the vaccine. Thus, in the question, we asked for their "Revaccination" intention. Even for the same individual, intentions varied according to circumstances. To obtain the key dependent variable, we asked which alphabetical column their childhood (or adulthood) surnames were located in. In our sample, approximately 60% of females changed their surname, compared to only 4% of males. This reflects traditional Japanese norms that favor the preservation of the male family line.

**Table 1:** Definition of variables used in estimations and their descriptive statistics.

Variables	Definition	Obs	Mean	s.d	max	min
Outcome variable						
*Revaccine*	Will you get a shot of vaccination? Choose from 5 choices where larger values show a higher intention to get the shot Respondent’s choices: 1, 2, 3, 4, 5	11,680	4.42	0.96	5	1
Surname						
*Name child*	Number of alphabetical columns of childhood surname initial ranges 1–10; 10 if respondent’s childhood surname initial is in “A” column (the first column). 1 if respondent’s surname initial is in “Wa” column (the 10th column)	11,680	7.11	2.45	10	1
*Name child_6_10*	1 if respondent’s childhood surname initial is in “A,” “,Ka”, “Sa”, “Ta”, “Na” column (the first to fifth columns), otherwise 0	11,680	0.70	0.45	1	0
*Name Adult*	Number of columns of adulthood surname initial ranges 1–10; 10 if respondent’s adulthood surname initial is in “A” column (the first column). 1 if respondent’s surname initial is in “Wa” column (the 10th column)	11,680	7.10	2.42	10	1
*Name Adult_6_10*	1 if respondent’s adulthood surname initial is in “A,” “Ka,” “Sa,” “Ta,” and “Na” column (the first to fifth columns), otherwise 0	11,680	0.70	0.46	1	0
*Name adult_1*	1 if respondent’s adulthood surname initial is in “A”, (the first column), otherwise 0	11,680	0.22	0.41	1	0
Primary school						
*Mixed-gender list*	1 if mixed-gender list is used in primary school, 0 otherwise Choose from 3 choices Respondent’s choices: 1 (yes), 2 (no), 3 (forget or will not respond)	5248	0.33	0.46	1	0
*Female teacher*	1 if class teacher is female at in the first grade in primary school, 0 otherwise Respondent’s choices: 1 (yes), 2 (no), 3 (forget or will not respond)	11,680	0.80	0.39	1	0
Control variables						
*Female*	1 if respondent is female, otherwise 0	11,680	0.49	0.50	1	0
*Ages*	Respondent’s ages	11,680	54.9	14.3	1	0
*University*	1 if respondent graduated from university, otherwise 0	11,680	0.45	0.50	1	0
*Damage*	How serious are your symptoms if you are infected with the novel coronavirus (an expectation of how bad an infection would be)? Choose from 6 choices 1 (very small influence) to 6 (death)	11,680	3.57	1.05	6	1
Job status						
* Office worker*	1 if respondent is office worker, otherwise 0	11,680	0.18	0.38	1	0
* Executive*	1 if respondent is company executives, otherwise 0	11,680	0.07	0.25	1	0
* Public officer*	1 if respondent is public officers, otherwise 0	11,680	0.05	0.22	1	0
* Self-employment*	1 if respondent is self-employed, otherwise 0	11,680	0.05	0.22	1	0
* Specialist*	1 if respondent is specialist, otherwise 0	11,680	0.03	0.17	1	0
* Contract employee*	1 if respondent is contract employee, otherwise 0	11,680	0.05	0.22	1	0
* Part-time*	1 if respondent is part-time worker, otherwise 0	11,680	0.10	0.32	1	0
* Student*	1 if respondent is student, otherwise 0	11,680	0.01	0.12	1	0
* Homemaker*	1 if respondent is homemaker, otherwise 0	11,680	0.22	0.41	1	0
* No-job*	1 if respondent does not have job, otherwise 0	11,680	0.20	0.39	1	0
* Other jobs*	1 if respondent has other jobs, otherwise 0	11,680	0.01	0.13	1	0
Household income						
* Income_1*	1 if respondent’s household income is below 1 million yens, otherwise 0	11,680	0.02	0.14	1	0
* Income_1.5*	1 if respondent’s household income is 1–1.99 million yens, otherwise 0	11,680	0.07	0.25	1	0
* Income_2.5*	1 if respondent’s household income is 2–2.99 million yens, otherwise 0	11,680	0.13	0.33	1	0
* Income_3.5*	1 if respondent’s household income is 3–3.99 million yens, otherwise 0	11,680	0.17	0.37	1	0
* Income_4.5*	1 if respondent’s household income is 4–4.99 million yens, otherwise 0	11,680	0.14	0.34	1	0
* Income_5.5*	1 if respondent’s household income is 5–5.99 million yens, otherwise 0	11,680	0.12	0.32	1	0
* Income_6.5*	1 if respondent’s household income is 6–6.99 million yens, otherwise 0	11,680	0.09	0.28	1	0
* Income_7.5*	1 if respondent’s household income is 7–7.99 million yens, otherwise 0	11,680	0.06	0.24	1	0
* Income_8.5*	1 if respondent’s household income is 8–8.99 million yens, otherwise 0	11,680	0.04	0.20	1	0
* Income_9.5*	1 if respondent’s household income is 9–9.99 million yens, otherwise 0	11,680	0.05	0.22	1	0
* Income_11*	1 if respondent’s household income is 10–11.99 million yens, otherwise 0	11,680	0.04	0.19	1	0
* Income_13.5*	1 if respondent’s household income is 12–14.99 million yens, otherwise 0	11,680	0.03	0.18	1	0
* Income_17.5*	1 if respondent’s household income is 15–19.99 million yens, otherwise 0	11,680	0.02	0.15	1	0
* Income_25*	1 if respondent’s household income is over 20 million yens, otherwise 0	11,680	0.01	0.11	1	0
*Time_Jan_2022*	1 if the data is gathered from14 to 19 January in 2022, otherwise 0	11,680	0.13	0.33	1	0
*Time_Feb_2022*	1 if the data is gathered from 25 February to 2 March in 2022, otherwise 0	11,680	0.13	0.33	1	0
*Time_Apr_2022*	1 if the data is gathered from 15 to 20 April in 2022, otherwise 0	11,680	0.13	0.33	1	0
*Time_May_2022*	1 if the data is gathered from 20 to 25 May in 2022, otherwise 0	11,680	0.13	0.33	1	0
*Time_June_2022*	1 if the data is gathered from 17 to 22 June in 2022, otherwise 0	11,680	0.13	0.33	1	0
*Time_July_2022*	1 if the data is gathered from 15 to 20 July in 2022, otherwise 0	11,680	0.13	0.33	1	0
*Time_Aug_2022*	1 if the data is gathered from 19 to 23 August in 2022, otherwise 0	11,680	0.13	0.33	1	0
*Time_Sep_2022*	1 if the data is gathered from 16 to 21 September in 2022, otherwise 0	11,680	0.13	0.33	1	0

During the sample collection process, some participants stopped taking the surveys and dropped out (Table 2). We limited the samples used for the analysis to respondents who participated in the 19th to 26th waves, allowing us to follow the same individuals. All individuals participated in the survey eight times. Table 1 shows 11,680 observations for most variables, indicating that 1,460 unique individuals were included in the sample. For a closer examination, we restricted the sample to those who answered questions about individual characteristics, including educational circumstances in primary school.

**Table 2:** Participants removal.

Number of surveys	Dates	Obs	Obs (completed the first vaccination)	Obs. (used for analysis)
1	March 13–16, 2020	4359 (7965)		
2	March 27–30, 2020	3495		
3	Apr. 10–13, 2020	4013		
4	May 8–11, 2020	3996		
5	June 12–15, 2020	3877		
6	Oct 23–28, 2020	3626		
7	Dec 4–8, 2020	3491		
8	Jan. 15–19, 2021	3509		
9	Feb. 17–22, 2021	3529		
10	Mar. 24–29, 2021	3440		
11	Apr. 23–26, 2021	3304		
12	May 28–31, 2021	3280		
13	June 25–30, 2021	3392		
14	July 30–Aug 4, 2021	3349		
15	Aug 27–Sep. 1, 2021	3347		
16	Sep 24–Sep. 29, 2021	3310		
17	Oct 29–Nov. 4, 2021	3284		
18	Nov 26–Dec. 1, 2021	3238		
19	Jan. 14–19, 2022	3210	2702	1460
20	Feb. 25–Mar 2, 2022	3183	2666	1460
21	Apr. 15–20, 2022	3113	2624	1460
22	May. 20–25, 2022	3040	2568	1460
23	Jun. 17–22, 2022	3014	2545	1460
24	Jul. 15–20, 2022	2971	2512	1460
25	Aug. 19–23, 2022	2971	2523	1460
26	Sep. 16–23, 2022	2925	2463	1460
Total		88,266	20,603	11,680

Numbers in parentheses indicate the subjects who were offered the first wave. Observations made prior to the 18th survey in the shaded areas were not included in the sample.

Childhood circumstances in school form non-cognitive skills that influence life outcomes^7,61–65^. We purposefully collected information about the hidden curriculum. To identify the childhood surname effect, we asked the participants about the alphabetical order of adult and childhood surnames. As shown in Table 1, the type of list and gender of teachers in the primary school were included as variables to capture educational circumstances. We could only gather 5248 observations about the type of list because many respondents did not remember it. Eventually, for closer examination, the number of respondents was reduced to 656. In Table 1, the mean of *Mixed-gender list* was 0.33, indicating that a mixed-gender list was used for 33% of respondents.

**Figure 1:**
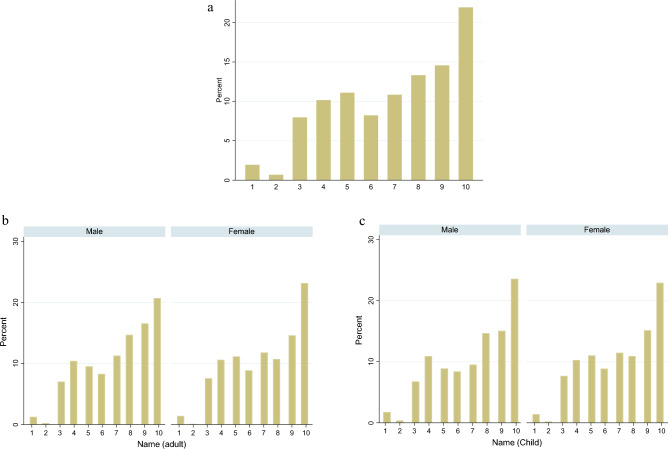
(**a**) Distribution of adulthood name orders using general population data. Data from the top 1500 family names were used to calculate percentages. It covers 93,200,000 individuals, accounting for approximately 75% of the Japanese population (12,570,000). Source: Family Name rankings. https://myoji-yurai.net/prefectureRanking.htm (accessed on December 18, 2022). (**b**) Distribution of adult name orders. (**c**) Distribution of childhood name orders. Name: For the convenience of interpretation, surnames earlier in the Japanese alphabetical list have larger values. To put it precisely, values 1, 2, 3, 4, 5, 6, 7, 8, 9 and 10 correspond to “Wa”, “Ra”, “Ya”, “Ma”, “Ha”, ”Na”, “Ta”, “Sa”, “Ka” and “A”.

The distribution of the surname orders in the general Japanese population is shown in Fig. 1a. For the convenience of interpretation of the estimation results in a later section, the earlier the surname, the larger the number of alphabetical columns. The earliest column “A” is around 22%, which is the highest ration among all columns. Using the data collected independently in this study, the distributions shown in Fig. 1b,c are similar to those in Fig. 1a. This indicates the representativeness of the data used in this study in terms of surname distribution. Furthermore, there were no differences between males and females or between childhood and adult names. Hence, name distribution is not biased by gender and is influenced by life events, such as marriage. We can appropriately compare the influence of surname order between males and females, as well as between childhood and adulthood.

### Ethical issues

Participants were informed of the purpose of the study and their right to quit at any time. All survey participants provided their consent to participate via the internet in an anonymous online survey by INTAGE, Inc. After agreeing to participate, they responded to the questions in the survey.

The authors did not obtain any personal information about the participants.

The survey design in this study was conducted with ex ante approval from the Ethics Committee of the Graduate School of Economics, Osaka University. Data collection was performed in accordance with the relevant guidelines and regulations. The ethics approval number of Osaka University for this study is R021014.

### Hypotheses

Apart from cognitive skills, non-cognitive skills generated during childhood improve various aspects of daily life^66–68^. These skills are often formed during early school and help individuals adapt to new phases of life. Even in adulthood, these noncognitive skills help individuals cope with challenging and unfamiliar situations. We hypothesized that having an early alphabetical surname might enhance school experience and consequently reduce the psychological cost of receiving vaccinations.

Over time, vaccines tend to become less effective, even after receiving a second dose of the COVID-19 vaccination. Therefore, revaccination is necessary to prevent the spread of COVID-19. Individuals who have already received a second shot may choose to undergo revaccination. Those whose surnames appeared early on the school register were less likely to view revaccination as troublesome. Hence, we propose Hypothesis 1:

*Hypothesis 1: Having childhood earlier surname is positively correlated with the intention for revaccination.*

Compared with males, females are less inclined to be “over-confident” or to take risk^69,70^. This is consistent with female health-related behavior, in that females are more cautious and gather far more health-related information than males when making health decisions^71^. In fact, females were less likely to receive a shot of the COVID-19 vaccine than males^35,72–74^. There seems to be more room for females to change their attitudes toward vaccination uptake through education. Therefore, learning experiences in childhood are thought to have a greater influence on females in unexpected situations, such as the COVID-19 pandemic. Furthermore, adopting a mixed-gender list is expected to increase opportunities for female students because they are less likely to suffer from unintended gender discrimination. Therefore, a mixed-gender list is expected to strengthen the correlation between surname and non-cognitive skills among female students. This leads us to propose *Hypothesis 2:*

*Hypotheses 2: Females with earlier surnames are more likely to show a positive attitude toward revaccination if the mixed gender list is used in their school.*

As discussed in the related literature, early surnames have various advantages. However, attitudes toward revaccination depend on noncognitive skills. Concerning non-cognitive skill formation, people with early surnames have an advantage in childhood and school life, but not in adulthood. Using a subsample of adults whose surnames have changed since childhood, we can identify the nature of the surname effect. This is because the influence of their current surname would not affect their non-cognitive skills, nor would it have affected their educational experiences. Hence, we propose *Hypothesis 3.*

Hypothesis 3: Adulthood surname is not correlated with the intention for revaccination.

### Methods

Our model assessed how surnames related to the outcome of the intention to revaccinate. We employ the following baseline model:

$$ Vaccine_{it} = \, \alpha_{0} + \, \alpha_{{1}} Name \, child\_6\_10 \, \left( {or \, Name \, child} \right)_{ih} + {\text{ X}}_{{\text{i}}} {\text{A }} + {\text{ e}}_{{\text{t}}} + {\text{ u}}_{{{\text{it}}}} \quad ({\text{model}}\,\,{1}) $$

where *Vaccine *_*it*_ is the outcome variable for individual *i* and time* h*. α denotes the regression parameters. The key variable was *Name child,* which ranged from 1 to 10. As described in Table 1, *Name child* is the number of alphabetical columns to which the respondent’s childhood surname initially belongs. For the convenience of interpretation, the larger the number, the earlier the surname was on the list. From *Hypothesis 1,* the coefficient of *Name child* is expected to have a positive sign. However, surnames were unlikely to correlate with the outcome variables. However, the correlation may not be linear. Therefore, instead of *Name child*, we include the indicator variable *Name child 6_10* in the alternative specifications. *Name child _6_10* is an indicator equal to one if the surname is in the first half of Column 6_10, otherwise 0.

X is a vector of the control variables. Several studies have found that teachers’ sex contributes to the formation of worldviews and preferences that influence healthy behaviors in adulthood^64,65,75^. The incentive for revaccination depends on the predicted damage of COVID-19 to health if one is infected by COVID-19. Furthermore, the subjective prediction of *Damage,* is likely to influence the attitude toward revaccination rather than objective prediction. We expected the coefficient of *Damage* to have a positive value because the larger the damage, the greater the motivation for revaccination. According to a survey article on COVID-19^76^, the following factors were correlated with views on COVID-19 vaccination: educational background, income level, occupation, sex, and age. Hence, we also incorporate university graduation, female sex, age, household income, and job status indicators. *e*_*t*_ captures the time-point effects when surveys are conducted. For instance, attitudes toward revaccination depend on the availability of vaccinations, degree of spread of COVID-19, and the implemented COVID-19 policy. These effects were controlled by including 7-time point indicators and a default time point of January 2022. *u*_*ih*_ is the error term.

First, the ordinary least squares (OLS) model was used. However, this distribution could not be considered normal. Alternative models were used for the estimations to check the robustness of the OLS results. The dependent variable was a Likert scale; therefore, we also conducted estimations using an Ordered Logit Model. Unlike in OLS, the degree of the effect of the key variable is difficult to interpret based on an Ordered Logit Model, although statistical significance can be checked. Therefore, instead of *Vaccine,* an indicator variable for vaccine acceptance was created as follows: the variable, *Vaccine 4_5,* is 1 if *Vaccine* is 4 or 5, otherwise 0. To interpret its effect appropriately, instead of the coefficient, the marginal effect is reported to show the effect of the surname on the probability of revaccination.

Various studies have found sex-based differences that correlate with precautionary behaviors. Owing to the male characteristics of overconfidence or a greater tendency to take risks^69,70^, males may be more likely to have a positive attitude toward revaccination. During the COVID-19 pandemic, people encounter not only the risk of contracting COVID-19 but also the risk of the side effects of vaccination. In the U.S., nearly 65% of people agreed with the statement “I am worried about the side effects of the vaccine for myself,” while approximately 40% agreed with the statement “the side effects of the vaccine are likely to be worse than COVID-19 itself”^77^. People are more inclined to hesitate to receive the COVID-19 vaccination if they consider the side effects to be more severe^20,76,78–83^. The learning effects in school may change females’ perceptions of being more positive about vaccination. To examine this, we use a specification that includes the interaction term between the female indicator and the key independent variable.

$$ Revaccine_{it} = \, \alpha \beta_{0} + \beta_{{1}} Name \, child \, 6\_10 \, \left( {or \, Name \, child} \right)_{ih} \times {\text{Female}} + \beta_{{2}} Name \, child \, 6\_10 \, \left( {or \, Name \, child} \right)_{ih} + {\text{ X}}_{{\text{i}}} {\text{B }} + {\text{ e}}_{{\text{t}}} + {\text{ u}}_{{{\text{it}}}} \quad \quad ({\text{model}}\,\,{2}) $$

β_1_ can be interpreted as a difference in the surname effect between male and female respondents. The expected positive sign of the coefficient of interaction term, β_1_, shows that females’ name order is more strongly correlated with their intention to be vaccinated compared with that of males’. We interpret β_2_ as suggesting a correlation between name order and intention to vaccinate for males. Furthermore, the specification, including the triple interaction term, was estimated to explore how the adoption of a mixed-gender list influenced name order and vaccination intention.

$$ \begin{gathered} Revaccine_{it} = \, \alpha \gamma_{0} + \gamma_{{1}} Name \, child\_6\_10 \, \left( {or \, Name \, child} \right)_{ih} \times Female \, \times Mixed - gender \, list + \gamma_{{2}} Name \, child\_6\_10 \, \left( {or \, Name \, child} \right)_{ih} \times Female \hfill \\ + \gamma_{{3}} Name \, child\_6\_10 \, \left( {or \, Name \, child} \right)_{ih} \times Mixed - gender \, list \hfill \\ + \gamma_{{4}} Female \, \times Mixed - gender \, list + \gamma_{{5}} Name \, child\_6\_10 \, \left( {or \, Name \, child} \right)_{ih} \hfill \\ + \gamma_{{6}} Female + \gamma_{{7}} Mixed - gender \, list + \gamma_{{8}} Name \, child\_6\_10 \, \left( {or \, Name \, child} \right)_{ih} \hfill \\ + {\text{ X}}_{{\text{i}}} {\text{G }} + {\text{ e}}_{{\text{t}}} + {\text{ u}}_{{{\text{it}}}} \quad \quad ({\text{model}}\,\,{3}) \hfill \\ \end{gathered} $$

Coefficient of Key variable is γ_1_, which shows how females who learned under a mixed-gender list are more likely to be positive for revaccination than males who also learned under a mixed gender list. Thus, it shows the degree to which the adoption of the mixed-gender list reinforces the correlation between name order and revaccination intention for females compared with males. Based on *Hypothesis 2,* the expected sign of γ_1_ is positive.

To identify the influence of the name order, we used a subsample of those whose childhood surnames differed from those in adulthood to compare the correlation between *Name child* and *Revaccine* with that between *Name adult* and *Revaccine*. The specification of the estimated function adds *Name adult* to the baseline model. We then simply compared the coefficient of *Name child* with that of *Name adult.* From *Hypothesis 3,* the coefficient of *Name child* is positive and its absolute value is larger than that of *Name adult*.

For the estimation, we used the statistical software Stata/MP 15.0.

## Estimation results

**Figure 2:**
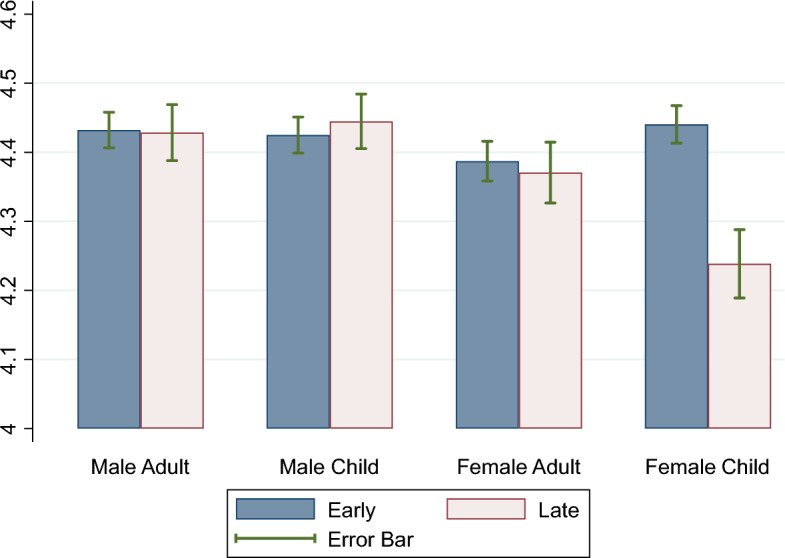
Intention to re-vaccinate early and late surnames. There are four pairs. The sample consisted of male and female participants. Furthermore, surnames are classified as early or later on the Japanese alphabetical list. Surname’s initial in “A,” “Ka,” “Sa,” “Ta” and “Na” is defined as “Early.” Surname’s initial in “Ha,” “Ma,” “Ya,” “Ra” and “Wa” is defined as “Late.”

Figure 2 demonstrates the degree of intention of revaccination by 8 groups using independently collected data. The sample was divided by sex and either early or late childhood surname order or adult name order. The surname category of the individuals changed according to whether their childhood surnames differed from their adult surnames. The degree of revaccination intention for males was higher than that for females, although the difference was not statistically significant. The "female-child" group on the left-hand side is of particular interest. Females with early childhood surnames were more likely to show higher values than those with later adulthood surnames, which was a statistically significant finding. Their vaccination intention was slightly higher than that of males, but the difference was not statistically significant. The findings in Fig. 2 imply that a correlation between surname order and vaccination intention was observed only for females’ childhood surnames. However, the various factors listed in Table 1 were not controlled for in Fig. 2. For a closer examination, we examined the regression estimation results.

The regression estimations are presented in Tables 3, 4, 5, 6, 7 and 8. Columns (1), (2), and (3) present the results using the OLS, Ordered Logit, and Logit models, respectively. In the OLS model, the coefficients are equivalent to the marginal effects. However, the Logit and Ordered Logit coefficients differ from the marginal effects and are difficult to understand. In the ordered logit model, the marginal effect can be obtained for each dependent variable (*vaccine*). For example, the effect of surname on the probability of *Vaccine* being 1. Similarly, we can obtain the surname effect when *Vaccine* are 2, 3, 4, and 5. In total, five marginal effects are needed to appropriately interpret the effect of *Vaccine.* In the logit model, we obtained only a marginal effect on the probability that *Vaccine* 4–5 are 1. Therefore, apart from CI 95%, coefficients are reported in columns (1) and (2), while marginal effects are reported in column (3)(In the results of the Logit model estimations in Tables 3, 4, 5, 6, 7 and 8, Stata 14 is used to calculate the marginal effect of the logit model as follows. Here, we assume “Y” is the binary dummy variable and so dependent variables. There are three independent variables.

1 $$ {\text{logit Y X1 X2 X3}} $$

2 $$ {\text{margins}},{\text{ dydx}}\left( {\text{X1 X2 X3}} \right) $$

In command (1), we can obtain only the coefficient and not the marginal effect. Therefore, directly after it, we added command (2) to calculate the marginal effect in the specification of command (1). Similarly, the marginal effects reported in column (3) have been calculated. For further details, see Supplementary “S2 Table Program”.). The marginal effect in the Logit model is expressed below.

$$\mathit{Pr}\left[Y=1\right|X]=F\left({\alpha }_{0}+{\alpha }_{1}X\right).$$

**Table 3:** Regression estimation baseline model: full sample.

	(1) OLS	(2) Ordered logit	(3) Logit
Panel A			
* Name child_6_10*	0.075*** (0.038 to 0.112)	0.145*** (0.058 to 0.231)	0.032*** (0.018 to 0.047)
* Female teacher*	− 0.04 (− 0.048 to 0.135)	0.055 (− 0.159 to 0.271)	0.016 (− 0.023 to 0.056)
* Ages*	0.020*** (0.016 to 0.023)	0.044*** (0.036 to 0.052)	0.007*** (0.005 to 0.008)
* Female*	− 0.001 (− 0.089 to 0.088)	− 0.061 (− 0.089 to 0.088)	− 0.009 (− 0.019 to 0.038)
* University*	0.054 (− 0.016 to 0.125)	0.153 (− 0.039 to 0346)	0.021 (− 0.002 to 0.044)
* Damage*	0.070*** (0.036 to 0.105)	0.185*** (0.110 to 0.260)	0.021*** (0.009 to 0.033)
* Office worker*	Default		
* Executive*	0.064 (− 0.135 to 0.264)	0.096 (− 0.382 to 0.575)	0.012 (− 0.051 to 0.076)
* Public officer*	0.005 (− 0.246 to 0.257)	0.085 (− 0.402 to 0.574)	0.006 (− 0.072 to 0.085)
* Self-employment*	0.015 (− 0.173 to 0.204)	0.062 (− 0.400 to 0.525)	0.001 (− 0.065 to 0.068)
* Specialist*	− 0.004 (− 0.304 to 0.397)	− 0.019 (− 0.699 to 0.659)	− 0.004 (− 0.098 to 0.088)
* Contract employee*	− 0.036 (− 0.243 to 0.172)	− 0.098 (− 0.559 to 0.362)	0.017 (− 0.048 to 0.082)
* Part-time*	0.036 (− 0.085 to 0.158)	0.142 (− 0.147 to 0.433)	0.018 (− 0.021 to 0.059)
* Student*	0.441** (0.053 to 0.830)	0.106*** (0.439 to 1.688)	0.132*** (0.097 to 0.166)
* Homemaker*	− 0.137 (− 0.319 to 0.044)	− 0.239 (− 0.620 to 0.141)	− 0.034 (− 0.093 to 0.024)
* No-job*	− 0.004 (− 0.154 to 0.145)	0.126 (− 0.223 to 0.476)	0.027 (− 0.018 to 0.074)
* Other jobs*	− 0.423* (− 0.859 to 0.012)	− 0.783** (− 0.155 to − 0.011)	− 0.119* (− 0.281 to 0.041)
* Income_1*	Default		
* Income_1.5*	0.087 (− 0.249 to 0.424)	0.285 (− 0.394 to 0.964)	0.051 (− 0.047 to 0.150)
* Income_2.5*	0.212 (− 0.152 to 0.576)	0.630 (− 0.246 to 0.257)	0.084 (− 0.008 to 0.176)
* Income_3.5*	0.235 (− 0.084 to 0.555)	0.644** (0.004 to 1.283)	0.101 (0.027 to 0.175)
* Income_4.5*	0.152 (− 0.186 to 0.491)	0.456 (− 0.230 to 1.144)	0.083 (0.001 to 0.166)
* Income_5.5*	0.162 (− 0.211 to 0.536)	0.523 (− 0.221 to 1.268)	0.079 (− 0.013 to 0.172)
* Income_6.5*	0.210 (− 0.182 to 0.603)	0.597 (− 0.210 to 1.405)	0.092 (0.006 to 0.178)
* Income_7.5*	0.121 (− 0.328 to 0.571)	0.437 (− 0.465 to 1.340)	0.083 (− 0.016 to 0.183)
* Income_8.5*	0.279 (− 0.134 to 0.693)	0.773* (− 0.063 to 1.610)	0.103* (0.207 to 0.178)
* Income_9.5*	0.285 (− 0.127 to 0.697)	0.735* (− 0.0.90 to 1.561)	0.107* (0.031 to 0.183)
* Income_11*	0.145 (− 0.200 to 0.491)	0.412 (− 0.270 to 1.094)	0.069 (− 0.012 to 0.152)
* Income_13.5*	0.443** (0.099 to 0.788)	1.284*** (0.539 to 2.029)	0.126*** (0.077 to 0.176)
* Income_17.5*	0.315 (− 0.242 to 0.874)	0.912 (− 0.367 to 2.193)	0.091 (− 0.008 to 0.191)
* Income_25*	− 0.091 (− 0.494 to 0.310)	0.002 (− 0.852 to 0.857)	− 0.023 (− 0.163 to 0.115)
* Time_Jan_2022*	Default		
* Time_Feb_2022*	− 0.025 (− 0.059 to 0.008)	− 0.064 (− 0.165 to 0.036)	− 0.013* (− 0.028 to 0.017)
* Time_Apr_2022*	− 0.108*** (− 0.143 to − 0.073)	− 0.280*** (− 0.374 to − 0.187)	− 0.044*** (− 0.060 to − 0.028)
* Time_May_2022*	− 0.190*** (− 0.237 to − 0.143)	− 0.469*** (− 0.570 to − 0.368)	− 0.072*** (− 0.089 to − 0.056)
* Time_June_2022*	− 0.275*** (− 0.313 to − 0.236)	− 0.667*** (− 0.749 to − 0.584)	− 0.090*** (− 0.106 to − 0.075)
* Time_July_2022*	− 0.232*** (− 0.274 to − 0.189)	− 0.579*** (− 0.672 to − 0.487)	− 0.087*** (− 0.104 to − 0.069)
* Time_Aug_2022*	− 0.241*** (− 0.284 to − 0.199)	− 0.565*** (− 0.659 to − 0.472)	− 0.089*** (− 0.107 to − 0.070)
* Time_Sep_2022*	− 0.279*** (− 0.326 to − 0.232)	− 0.636*** (− 0.747 to − 0.526)	− 0.102*** (− 0.119 to − 0.084)
R-square	0.12		
Pseudo R-square		0.06	0.10
Observations	11,680	11,680	11,680
Panel B			
*Name child*	0.022*** (0.015 to 0.029)	0.055*** (0.039 to 0.072)	0.009*** (0.006 to 0.011)
R-square	0.12		
Pseudo R-Square		0.06	0.10
Observations	11,680	11,680	11,680

Numbers within parentheses are 95% confidence intervals using robust standard errors in columns (1) and (2) and the delta method standard errors in column (3). Numbers within parentheses are coefficients for the OLS and Ordered Logit models in columns (1) and (2). The marginal effects for the Logit model are presented in column (3). All the control variables are listed in Table 3. The Standardized EXCESS RATE is (rate of the group’s population–rate of the general population)/ rate of the general population.

*p < 0.10, **p < 0.5, ***p < 0.01.

**Table 4:** Regression estimation.

	(1) OLS	(2) Ordered logit	(3) Logit
Panel A			
*Name child_6_10* × *female*	0.205*** (0.131 to 0.280)	0.465*** (0.291 to 0.639)	0.082*** (0.054 to 0.111)
*Name child_6_10*	− 0.023 (− 0.073 to 0.024)	− 0.094 (− 0.218 to 0.029)	− 0.011 (− 0.032 to 0.009)
*Female*	− 0.144*** (− 0.211 to − 0.077)	− 0.390*** (− 0.547 to − 0.232)	− 0.066*** (− 0.092 to − 0.407)
Control variables in Table 3	Yes	Yes	Yes
R-square	0.12		
Pseudo R-square		0.06	0.10
Observations	11,680	11,680	11,680
Panel B			
* Name child* × *female*	0.027*** (0.013 to 0.041)	0.064*** (0.008 to 0.120)	0.012*** (0.007 to 0.017)
* Name child*	0.008* (− 0.001 to 0.018)	0.022* (− 0.001 to 0.046)	0.002 (− 0.002 to 0.006)
* Female*	− 0.195*** (− 0.304 to − 0.086)	− 0.511*** (− 0.760 to − 0.262)	− 0.099*** (− 0.136 to − 0.056)
Control variables in Table 3	Yes	Yes	Yes
R-square	0.12		
Pseudo R-square		0.06	0.10
Observations	11,680	11,680	11,680

Numbers within parentheses are 95% confidence intervals using robust standard errors in columns (1) and (2) and the delta method standard errors in column (3). Numbers within parentheses are coefficients for the OLS and Ordered Logit models in columns (1) and (2). The marginal effects for the Logit model are presented in column (3). All the control variables are listed in Table 3.

Adding interaction terms between surname order and female indicator.

*p < 0.10, **p < 0.5, ***p < 0.01.

**Table 5:** Regression estimation.

	(1) OLS	(2) Ordered logit	(3) Logit
Panel A			
* Name child_6_10* × *Female* × *mixed-gender list*	0.760*** (0.525 to 0.996)	1.473*** (0.900 to 0.204)	0.301*** (0.204 to 0.398)
* Name child_6_10* × *Female*	0.120* (− 0.015 to 0.256)	0.453*** (0.124 to 0.781)	0.015 (− 0.380 to 0.069)
* Name child_6_10* × *Mixed-gender list*	− 0.469*** (− 0.623 to − 0.314)	− 1.063*** (− 6_1006 to − 0.620)	− 0.198*** (− 0.277 to − 0.119)
* Name child_6_10*	0.024 (− 0.075 to 0.123)	− 0.081 (− 0.331 to 0.169)	0.009 (− 0.032 to 0.050)
* Female* × *mixed-gender list*	− 0.628*** (− 0.826 to − 0.430)	− 1.359*** (− 1.841 to − 0.877)	− 0.216*** (− 0.300 to − 0.132)
* Female*	− 0.044 (− 0.171 to 0.081)	− 0.326** (− 0.622 to − 0.30)	− 0.035 (− 0.083 to 0.069)
* Mixed-gender list*	0.235*** (0.116 to 0.354)	0.604*** (0.223 to 0.984)	0.101*** (0.031 to 0.171)
Control variables in Table 3	Yes	Yes	Yes
R-square	0.16		
Pseudo R-square		0.08	0.10
Observations	5248	5248	5248
Panel B			
* Name child* × *female* × *mixed-gender list*	0.132*** (0.086 to 0.177)	0.237*** (0.130 to 0.344)	0.045*** (0.028 to 0.062)
* Name child* × *female*	0.026** (0.001 to 0.053)	0.093*** (0.030 to 0.156)	0.008 (− 0.001 to 0.018)
* Name child* × *mixed-gender list*	− 0.063*** (− 0.095 to − 0.031)	− 0.131*** (− 0.209 to − 0.053)	− 0.019*** (− 0.031 to − 0.007)
* Name child*	0.007 (− 0.012 to 0.028)	0.009 (− 0.039 to 0.058)	− 0.001 (− 0.008 to 0.006)
* Female* × *mixed-gender list*	− 1.1003*** (− 1.345 to − 0.662)	− 1.911*** (− 2.678 to 1.144)	− 0.301*** (− 0.426 to − 0.176)
* Female*	− 0.165 (− 0.372 to 0.041)	− 0.693*** (− 1.167 to − 0.219)	− 0.090** (− 0.165 to − 0.014)
* Mixed-gender list*	0.356*** (0.129 to 0.582)	0.746** (0.171 to 1.321)	0.087* (− 0.006 to 0.182)
Control variables in Table 3	Yes	Yes	Yes
R-square	0.16		
Pseudo R-square		0.08	0.10
Observations	5248	5248	5248

Numbers within parentheses are 95% confidence intervals using robust standard errors in columns (1) and (2) and the delta method standard errors in column (3). Numbers within parentheses are coefficients for the OLS and Ordered Logit models in columns (1) and (2). The marginal effects for the Logit model are presented in column (3). All the control variables are listed in Table 3.

Adding triple interaction terms.

*p < 0.10, **p < 0.5, ***p < 0.01.

**Table 6:** Regression estimation.

	(1) OLS	(2) Ordered logit	(3) Logit
Panel A			
* Name child_6_10*	0.174*** (0.106 to 0.242)	0.338*** (0.177 to 0.498)	0.074*** (0.048 to 0.099)
* Name adult_6_10*	− 0.062* (− 0.128 to 0.002)	− 0.161* (− 0.328 to 0.005)	− 0.018 (− 0.046 to 0.010)
Control variables in Table 3	Yes	Yes	Yes
R-square	0.09		
Pseudo R-square		0.08	0.09
Observations	3640	3640	3640
Panel B			
* Name child*	0.039*** (0.027 to 0.051)	0.094*** (0.065 to 0.124)	0.016*** (0.011 to 0.020)
* Name adult*	− 0.001 (− 0.013 to 0.012)	− 0.011 (− 0.033 to 0.031)	− 0.001 (− 0.007 to 0.03)
Control variables in Table 3	Yes	Yes	Yes
R-square	0.09		
Pseudo R-square		0.08	0.09
Observations	3408	3408	3408

Numbers within parentheses are 95% confidence intervals using robust standard errors in columns (1) and (2) and the delta method standard errors in column (3). Numbers within parentheses are coefficients for the OLS and Ordered Logit models in columns (1) and (2). The marginal effects for the Logit model are presented in column (3). All the control variables are listed in Table 3.

Comparison of childhood and adult surnames. Subsample of childhood surnames differing from adult surnames. Male and Female sample.

*p < 0.10, **p < 0.5, ***p < 0.01.

**Table 7:** Regression estimation.

	(1) OLS	(2) Ordered logit	(3) Logit
Panel A			
* Name child_6_10*	0.198*** (0.127 to 0.269)	0.379*** (0.214 to 0.545)	0.087*** (0.061 to 0.113)
* Name adult_6_10*	− 0.101*** (− 0.168 to 0.035)	− 0.228** (− 0.400 to − 0.055)	− 0.025* (− 0.054 to 0.003)
Control variables in Table 3	Yes	Yes	Yes
R-square	0.09		
Pseudo R-square		0.08	0.09
Observations	3640	3640	3640
Panel B			
* Name child*	0.045*** (0.032 to 0.057)	0.108*** (0.078 to 0.139)	0.019*** (0.013 to 0.023)
* Name adult*	− 0.009 (− 0.025 to 0.004)	− 0.016 (− 0.050 to 0.016)	− 0.003 (− 0.008 to 0.001)
Control variables in Table 3	Yes	Yes	Yes
R-square	0.09		
Pseudo R-square		0.08	0.09
Observations	3408	3408	3408

Numbers within parentheses are 95% confidence intervals using robust standard errors in columns (1) and (2) and the delta method standard errors in column (3). Numbers within parentheses are coefficients for the OLS and Ordered Logit models in columns (1) and (2). The marginal effects for the Logit model are presented in column (3). All the control variables are listed in Table 3.

Comparison of childhood and adult surnames. Subsample of childhood surnames differing from adult surnames. Female sample.

*p < 0.10, **p < 0.5, ***p < 0.01.

**Table 8:** Regression estimation for checking non-linearity.

	(1) OLS	(2) Ordered logit	(3) Logit
Panel A: Male and female sample			
*Name child_1*		< Default >	
*Name child_2*	− 0.333 (− 0.764 to 0.096)	− 0.683 (− 1.616 to 0.250)	− 0.125 (− 0.278 to 0.026)
*Name child_3*	− 0.0114 (− 0.372 to 0.143)	− 0.179 (− 0.848 to 0.489)	− 0.009 (− 0.104 to 0.085)
*Name child_4*	− 0.120 (− 0.355 to 0.114)	− 0.380 (− 0.100 to 0.242)	− 0.030 (− 0.119 to 0.588)
*Name child_5*	− 0.078 (− 0.327 to 0.171)	− 0.181 (− 0.833 to 0.470)	0.0003 (− 0.091 to 0.091)
*Name child_6*	0.047 (− 0.198 to 0.293)	− 0.024 (− 0.670 to 0.621)	0.067 (− 0.028 to 0.162)
*Name child_7*	− 0.167 (− 0.412 to 0.077)	− 0.501 (− 1.139 to 0.136)	− 0.040 (− 0.132 to 0.051)
*Name child_8*	0.110 (− 0.123 to 0.344)	0.053 (− 0.576 to 0.682)	0.064 (− 0.026 to 0.155)
*Name child_9*	0.121 (− 0.113 to 0.355)	0.219 (− 0.415 to 0.853)	0.098** (0.005 to 0.171)
*Name child_10*	0.156 (− 0.068 to 0.381)	0.480 (− 0.137 to 0.109)	0.083* (− 0.004 to 0.171)
Control variables in Table 3	Yes	Yes	Yes
R-square	0.09		
Pseudo R-square		0.08	0.09
Observations	3640	3640	3640
Panel B: female sample			
*Name child_1*		<Default>	
*Name child_2*	− 0.408 (− 0.995 to 0.178)	− 0.597 (− 1.562 to 0.366)	− 0.181 (− 0.384 to 0.020)
*Name child_3*	− 0.154 (− 0.413 to 0.104)	− 0.234 (− 0.908 to 0.438)	− 0.020 (− 0.116 to 0.074)
*Name child_4*	− 0.143 (− 0.378 to 0.091)	− 0.390 (− 1.018 to 0.237)	− 0.047 (− 0.136 to 0.042)
*Name child_5*	− 0.061 (− 0.312 to 0.188)	− 0.110 (− 0.770 to 0.549)	0.011 (− 0.081 to 0.104)
*Name child_6*	0.030 (− 0.215 to 0.275)	− 0.058 (− 0.707 to 0.590)	0.066 (− 0.029 to 0.163)
*Name child_7*	− 0.129 (− 0.372 to 0.114)	− 0.423 (− 1.064 to 0.216)	− 0.024 (− 0.117 to 0.068)
*Name child_8*	0.083 (− 0.150 to 0.316)	0.023 (− 0.610 to 0.657)	0.058 (− 0.032 to 0.150)
*Name child_9*	0.120 (− 0.113 to 0.354)	0.247 (− 0.390 to 0.886)	0.106** (0.011 to 0.197)
*Name child_10*	0.192* (− 0.031 to 0.415)	0.619* (− 0.003 to 1.243)	0.090** (0.007 to 0.185)
Control variables in Table 3	Yes	Yes	Yes
R-square	0.09		
Pseudo R-square		0.08	0.09
Observations	3408	3408	3408

The coefficient of X is ${\alpha }_{1}$. In addition to these coefficients, a marginal effect can be obtained at $X$,

$$\frac{\partial \mathit{Pr}\left[Y=1\right|X]}{\partial x}=[{\alpha }_{1}]\times F{\prime}({\alpha }_{0}+{\alpha }_{1}X)$$

The value of *X* varies from minimum to maximum values if *X* is a linear variable. Therefore, we can calculate various marginal effects according to the value of *X*. In column (3), we reported the estimated marginal effect at the mean value of *X*.

In Panel A of Tables 3, 4, 5, 6 and 7, the surname indicator variable *Name Child* _6_10 is used to capture the surname effect. In PANEL B, *Name child* is used. The effect of surname order might not be linear. For testing it, Table 8 reports results where key variables are 9 indicators for columns “Ya”-“A” (2–10) and column ”Wa” (1) is the default.

Table 3 shows, as is expected, *child_6_10* in PANELA and *Name child* in PANEL B while being statistically significant at the 1% point. Therefore, *Hypothesis 1* is supported. To consider the degree of the surname effect, we observe the result of the Logit model in Panel A. As for control variables in Panel A, *University*, *Female*, household income indicators and job status indicators are not statistically significant, which is not congruent to previous works^76^. In the dataset, in order to consider revaccination intention, all participants have been vaccinated. These characteristics might be correlated with whether people are vaccinated, but not with whether vaccinated people intend to be revaccinated. However, several characteristics are correlated with the intention for revaccination. Consistent with existing works^82,84^, significant positive sign of *Ages* can be interpreted as suggesting that benefit from revaccination is larger than its cost because aged people are more likely to be infected with COVID-19. The effect seems to be driven more by the fact that if they get it, they are likely to have severe cases or die. In line with Kreps^82^ *Damage* shows the significant positive sign, implying that predicted seriousness of COVID-19 gives individuals incentive to revaccinate.

As shown in Panels A and B in Table 4, the significant positive sign of the interaction terms implies that the correlation between surname order and revaccination intentions is stronger for females than for males.

Table 5 shows the results of the triple interaction term to examine the impact of the mixed gender list. In both TABLES A and B, the triple interaction term indicates a positive sign and a statistically significant 1% level in all columns. Hence, the results are robust to alternative specifications. The correlation between early surname and revaccination intention is greater for females when a mixed-gender list is used in their school than when a mixed-gender list is not used. This result leads us to argue that the adoption of a mixed gender list strengthened early name influence for females. Females with early surnames are more likely to be vaccinated than males with early surnames. Furthermore, the difference was larger by 30% when a mixed-gender list was used than when the list was not used. The effect of the mixed gender list is sufficiently sizable to widen the difference in the surname effect between genders.

This is consistent with the *Hypothesis 2.*

In Table 6, to compare childhood surname impact with the adulthood one, *Name adult_6_10* and *Name adult* were included in PANEL A and PANEL B, respectively. Furthermore, the estimation was conducted using a subsample of those whose childhood surnames differed from those in their adulthood. Women generally change their surnames after marriage, whereas men do not. Thus, males in the subsample can be considered different from the general male population. Hence, for the estimation, we also used a subsample excluding males, and the results are reported in Table 7. *Name Child_6_10* and *Name Chinld* indicate significant positive signs in all columns. Meanwhile, for *Name adult_6_10* and *Name adult,* we did not observe a positive sign in any column. These results imply that the surname order during childhood leads to differences in revaccination intentions. This effect was sizable and larger than that shown in Table 3. This finding, as indicated in Tables 6 and 7, strongly supports *Hypothesis 3.*

Table 8 shows results using nine indicator variables to investigate the childhood surname effect. In Columns (1) and (2), we hardly observed statistical significance. In column (3), the results of the Logit estimation show a positive sign while having statistical significance for *Name child_9* and *Name child_10*. This means that the respondents with names in the first column of the list are more likely to be vaccinated than those with names in the last column of the list. This is consistent with the results in Table 3, although the results vary according to estimation models.

## Discussion

### Implication

This study aimed to examine how surname orders form non-cognitive skills that influence gender differences in revaccination intentions. Additionally, we examined the effect of the educational policy of adopting a mixed-gender list on long-term health behaviors, such as revaccination.

The estimation results can be interpreted from the viewpoint of a cost–benefit analysis. Guaranteed payments for COVID-19 vaccination have increased vaccination rates^18^. Therefore, people are hesitant about vaccination if the cost is greater than the benefits. In other words, the benefits outweigh the costs for those receiving a vaccination. The vaccinated individuals agreed to the benefits of the vaccination when they received at least the first and second shots. However, their attitudes towards revaccination varied. One reason is that the subjective and psychological costs of revaccination differ.

Many studies have found that females are hesitant about being vaccinated against COVID-19^35,72–74^. Reference^7^ found that informal school education contributes to the promotion of preventive behaviors against COVID-19. However, it is unknown how the sex gap in vaccination intentions can be reduced. This study provides evidence that insufficient experience during new phases of school makes females’ reluctant to revaccinate.

To mitigate the surname effect, teachers must randomly appoint students. However, it is necessary for teachers to remember the students who have already been appointed and then appoint other students. However, this increases teachers’ burden. Furthermore, teachers’ appointments are thought to reflect their preferences, leading to favoritism. Artificial intelligence (AI) may be useful for teachers. Setting up an AI device to randomly assign students reduces teachers’ burden and avoids favoritism.

### Strength

Various studies have considered the influence of name order. However, this study is the first to analyze the outcome of name order by bridging educational economics and public health. The name order list was used as part of the hidden curriculum, resulting in gender discrimination. A mixed-sex list has been adopted since 1980. This study showed that a mixed-gender list in schools reduces the gender gap in adulthood by considering revaccination intentions. A mixed-gender list is useful for forming noncognitive skills among females, and its influence persists even when they become adults.

### Limitation

We assumed that the surname was given exogenously; therefore, the setting was a natural experiment. However, the effects of surnames may have been inherited from one generation to another. The distribution of surnames among the elite and underclass indicated that social status was inherited^85^. Surnames that reflect inherited physical aptitudes are advantageous for certain sports^86,87^. Owing to data limitations, it was difficult to distinguish inherited effects from learning experiences in schools.

We assume that the adoption of a mixed-gender list is determined exogenously. However, the adoption of such a list may also affect teacher quality. If this holds true, then the causality is unclear. Consequently, we use a mixed-gender list as a proxy for teacher quality. Female teachers are less likely to discriminate against students of the same gender than male teachers. We controlled for the gender of the homeroom teachers. To a certain extent, teacher quality was controlled, although we aim to control teacher quality more precisely in future studies. Recall bias may occur when considering the type of list used in primary school, but in the questionnaire, respondents could choose “Forget or will not respond,” which mitigates this bias.

Although the additional contribution is minor for examining general COVID-19 vaccination intention, as numerous studies have already scrutinized it, we confined our sample to those who completed the first and second shots of the COVID-19 vaccination to consider intentional revaccination. Naturally, the estimation results may suffer from a selection bias. However, as of March 2023, the vaccination rate was approximately 80%, implying that the effect of selection bias was likely small^37^. Considering the policy implications when vaccinated people comprise the majority, using a subsample is sufficiently valuable.

As we showed, the rates for third and fourth shots were approximately 68%. Considering the mean of the revaccination variable, this value was lower than expected. This indicates that there are potential gaps between revaccination intentions and behaviors. One possible explanation for this is the time lag between the two. For example, active workers may be too busy to receive the shots immediately. If this holds true, the revaccination rate increases over time. However, during 2023, the third-shot revaccination rate has been slightly less than 70% and has hardly changed. Considering the real situation, before getting shots, COVID-19 was almost resolved, and the intention to get a shot was reduced drastically. However, this is only our speculation. This should be explored in the future, using datasets.

As explained in Section "Data collection". Data collection, “All respondents used the Internet to participate in the survey.” The elderly are less able to use the Internet because of cognitive decline, causing selection bias. Most estimation results consistently supported the hypotheses. However, the results obtained using the nine indicator variables reported in Table 8 were not robust. Therefore, future studies should pay careful attention to this issue.

## Conclusion

Surnames are given exogenously, and collecting surname data allows researchers to conduct quasi-experiments. The surname effect has been considered in various fields, including academic performance, election outcomes, and firm performance. The contributions of this study are as follows:

First, no study has analyzed the connection between the role of surnames in schools and non-educational outcomes in later life. This study is the first to compare childhood and adulthood surname orders to identify the critical role of surname order in forming non-cognitive skills.

Second, educational researchers and mass media have asserted that mixed-gender lists should be adopted to equalize learning opportunities between genders in school life^1–5^. In response to this claim, the rate of adoption of mixed gender lists has increased to 80% in primary and junior high schools. However, no studies have quantitatively verified the influence of adopting a mixed-sex list. We contribute to education research by showing the long-term effects of adopting a mixed-gender list in schools when unexpected events such as the COVID-19 pandemic occur.

The major finding is that gender differences are partly due to traditional name-order lists in schools. This list leads teachers to unintentionally discriminate against female students by reducing their learning experience. Insufficient female experience in school increases the psychological cost of revaccination, lowers its benefits, and delays the eradication of COVID-19.

While we provide evidence that a mixed-gender list is effective in reducing the gender gap in school experiences, this study deals with a very specific issue and derives a general argument. Thus, exploring the impact of mixed-gender lists on broader socioeconomic issues is crucial. These concerns should be addressed in future studies. Further examination of how childhood surname order influences the quality of adult life is warranted.

### Supplementary Information

Supplementary Information 1.

Supplementary Information 2.

Supplementary Information 3.

Supplementary Information 4.

## Data Availability

The datasets and codes for the statistical analysis in the current study are available as supplementary files.
