# Supplementary material for: Surname order and revaccination intentions during the COVID-19 pandemic
Source: Sci Rep. 2024 Feb 27;14:4750. doi: 10.1038/s41598-024-55543-2 (PMC10899220; doi:10.1038/s41598-024-55543-2)
Supplement: Supplementary file 1 — Supplementary Information 1. [file 41598_2024_55543_MOESM1_ESM.pdf]

Survey questions and answer options

(The English translation is shown first, followed by the Japanese version below.)

The variables for vaccination experience are based on the answers to the following questions.

	A5 I would like to ask you about the vaccination (including the third booster) against the novel coronavirus that is being offered in Japan. This vaccine will be provided free of charge. Please select "1" if you will never be vaccinated and "5" if you will definitely be vaccinated. If you have already been vaccinated, please think back to how you felt before you were vaccinated. 1：Never vaccinate ← →5：Never vaccinate
	Would you get the vaccine if it were available for free?
1	1
2	2
3	3
4	4
5	5

QUESTION ABOUT FAMILY NAME: Please select one of the options below that applies to you.

	C6 Please indicate the line in the "syllabary" of your current last name. Also, please indicate the first syllable of your surname when you were in the first grade of elementary school. (e.g. Abe or Ito would be on the "a" line, Sato or Suzuki would be on the "sa" line, and those with a muddled or semi-muddled sound, please indicate the "syllabic" line with the muddled sound removed).
	The "syllabary" of the current surname
1	"a" column or row of the kana syllabary
2	"ka" column or row of the kana syllabary
3	"sa" column or row of the kana syllabary
4	"ta" column or row of the kana syllabary
5	classification for Japanese verb with the dictionary form ending in "nu
6	"ha" column or row of the kana syllabary
7	classification for Japanese verb with the dictionary form ending in "mu
8	"ya" column or row of the kana syllabary
9	"ra" column or row of the kana syllabary
10	"wa" column or row of the kana syllabary
	The "syllabary" of my first grade last name
1	"a" column or row of the kana syllabary
2	"ka" column or row of the kana syllabary
3	"sa" column or row of the kana syllabary
4	"ta" column or row of the kana syllabary
5	classification for Japanese verb with the dictionary form ending in "nu
6	"ha" column or row of the kana syllabary
7	classification for Japanese verb with the dictionary form ending in "mu
8	"ya" column or row of the kana syllabary
9	"ra" column or row of the kana syllabary
10	"wa" column or row of the kana syllabary

Question of elementary school experience.

Question about mixed roster system (if the answer was "not applicable," the mixed roster system was defined as being used).

	When I was in the early grades of elementary school, for the most part, the order of attendance numbers was male first, female second.
1	apply (a rule)
2	not applicable
3	I don't remember.

Gender of homeroom teacher

	Was your first grade homeroom teacher a woman?
1	Yes, sir.
2	No, sir.
3	I don't remember.

Questions regarding gender, age, and education.

	B1 Please indicate your gender.
1	masculine gender
2	female
	B2 Please indicate your age.
	B3 Please indicate the last school from which you and your parents graduated. If you are currently enrolled in school, please indicate the school you are currently attending. Please choose one that applies.
	you
1	Graduated from elementary or junior high school (including elementary or senior high school)
2	High school graduate (including old junior high school, girls' school, vocational school, and teacher's training school) (including prospective graduates)
3	Junior college graduates (including colleges of technology) (including prospective graduates)
4	University Graduate (including old high school and old technical college) (including prospective graduates)
5	Completion of a master's degree (including prospective graduates)
6	Graduate School Doctoral Course Completed (including prospective graduates)
7	I don't want to answer.

A hypothetical question about the medical condition of a patient with new coronas.

	A7 If you were infected with the novel coronavirus, how sick do you think you would be?
1	Not much of an impact.
2	Takes 2–3 days to recover.
3	Takes about a week to recover.
4	Takes about a month to recover.
5	Severe enough to cause permanent injury.
6	Become seriously ill with the possibility of death.

Questions about your profession

Please select one of the options below that applies to you.

	[Attribute] Occupation
1	company employee
2	Company executives and managers
3	Civil servants and organization employees
4	independent business
5	Liberal and professional
6	Temporary and contract employees
7	Part-time job
8	Elementary school students and under
9	junior high school student
10	senior high school student
11	prep school (ronin year) student
12	Vocational school students, junior college students, college students, graduate students
13	Housewife/Househusband
14	without an occupation
15	Other

Question about annual household income.

Please select one of the options below that applies to you as your household income.

	[Attribute] Annual household income including tax
1	Less than 1 million yen
2	Less than 2 million yen
3	Less than 3 million yen
4	Less than 4 million yen
5	Less than 5 million yen
6	Less than 6 million yen
7	Less than 7 million yen
8	Less than 8 million yen
9	Less than 9 million yen
10	Less than 10 million yen
11	Less than 12 million yen
12	Less than 15 million yen
13	Less than 20 million yen
14	More than 20 million yen
15	I don’t know.
16	I don’t want to answer.

Survey questions and answer options

(最初に英訳、その下に日本語版を示している。)

ワクチン接種経験の変数については、下記の質問の回答に基づいている。

	A5 日本で行われている新型コロナウイルスに対するワクチン接種(3 回目のブースターを含む)についてお伺いします。このワクチンは無料で提供される予定ですが、あなたは、ワクチンが受けられることになったら接種しますか。絶対に接種しない場合には“1”を、絶対に接種する場合には“5”をお選びください。すでに接種した人は接種する前の気持ちを思い出してお答えください。 1:絶対に接種しない← →5:絶対に接種する
	無料でワクチンが受けられることになったら接種しますか？
1	1
2	2
3	3
4	4
5	5

家族名に関する質問：あなたに当てはまる選択肢を下記の中から一つ選択してください。

	C6 あなたの現在の名字の「五十音」での行をお答えください。また、あなたの小学校 1 年生の頃の名字の「五十音」での行をお答えください。（例：阿部や伊藤なら「ア」行、佐藤や鈴木なら「サ」行、濁音・半濁音の方は、濁音を取った際の「五十音」をお答えください。）
	現在の名字の「五十音」
1	ア行
2	カ行
3	サ行
4	タ行
5	ナ行
6	ハ行
7	マ行
8	ヤ行
9	ラ行
10	ワ行
	小学校 1 年生の頃の名字の「五十音」
1	ア行
2	カ行
3	サ行
4	タ行
5	ナ行
6	ハ行
7	マ行
8	ヤ行
9	ラ行
10	ワ行

小学生時代の経験の質問。

混合名簿方式についての質問（「当てはまらない」と回答した場合、混合名簿方式を採用していると定義した）。

	小学校の低学年の頃、大半の期間は、出席番号順は男が先で、女が後だった
1	あてはまる
2	あてはまらない
3	覚えていない

担任教師の性別

	小学校１年生の担任の先生は女性でしたか？
1	はい
2	いいえ
3	覚えていない

性別、年齢、学歴に関する質問。

	B1 あなたの性別をお答えください。
1	男性
2	女性
	B2 あなたの年齢をお答えください。
	B3 あなたとあなたの両親が最後に卒業された学校をお答えください。在学中の方は、現在在学している学校をお答えください。あてはまるものを 1 つお選びください。
	あなた
1	小中学校 卒業（尋常小学校、高等小学校を含む）
2	高等学校 卒業（旧制中学校、女学校、実業学校、師範学校を含む）（卒業見込みを含む）
3	短期大学 卒業（高専等を含む）（卒業見込みを含む）
4	大学 卒業（旧制高校、旧制高等専門学校を含む）（卒業見込みを含む）
5	大学院修士課程 修了（卒業見込みを含む）
6	大学院博士課程 修了（卒業見込みを含む）
7	答えたくない

新型コロナに罹患した場合の病状についての仮想質問。

	A7 あなたが新型コロナウィルスに感染した場合、どのくらいの病状になると思いますか。1 つお選びください。
1	たいした影響はない
2	回復までに 2～3 日かかる
3	回復までに 1 週間ほどかかる
4	回復までに 1 ヶ月ほどかかる
5	後遺症が残るほどに重篤になる
6	死亡する可能性がある重篤な病気になる

職業についての質問

あなたに当てはまる選択肢を下記の中から一つ選択してください。

	[属性]職業
1	会社員
2	会社役員･管理職
3	公務員･団体職員
4	自営業
5	自由業･専門職
6	派遣･契約社員
7	パート･アルバイト
8	小学生以下
9	中学生
10	高校生
11	予備校生
12	専門学校生･短期大学生･大学生･大学院生
13	専業主婦･専業主夫
14	無職
15	その他

年間世帯年収についての質問。

世帯年収として、あなたに当てはまる選択肢を下記の中から一つ選択してください。

	[属性]世帯税込年収
1	100 万円未満
2	200 万円未満
3	300 万円未満
4	400 万円未満
5	500 万円未満
6	600 万円未満
7	700 万円未満
8	800 万円未満
9	900 万円未満
10	1000 万円未満
11	1200 万円未満
12	1500 万円未満
13	2000 万円未満
14	2000 万円以上
15	分からない
16	答えたくない

/*description of variables*/

Wave: Nubmer of surveys
Vacci: Revaccine
samp_vac2: Sample that completed the first and second shots.
iage: ages at the initial period.
name_chi: Name child
name_chi_6_10: Name child_6_1-
name_adu : Name adult
name_adu_6_10 Name adult_6_1-
mix_list: Mixed-gender list indicator
femal : female indicator
ftch_pri :Female teacher
univ: University graduate indicator
damage :Damage
i.work_dm :Job status
i.incom_dm : income level

/*table 1*/
xi:tabstat vacci iage name_chi name_chi_6_10 name_adu name_adu_6_10 mix_list femal ftch_pri univ damage i.work_dm i.incom_dm if balance_6_26==21 & wave>=19 & samp_vac2==1 & ftch_pri!=., stat(mean sd max min count)

/*table 2*/
tabstat wave , stat(count) by(wave)
tabstat wave if wave>=19 & samp_vac2==1 , stat(count) by(wave)

tabstat wave if balance_6_26==21 & wave>=19 & samp_vac2==1 & ftch_pri!=., stat(count) by(wave)

/*tab2*/

/*子供名のみ*/

/*職業、所得コントロール*/

/*全体*/

```
/*linear name_order*/
reg vacci name_chi iage  femal ftch_pri univ damage i.work_dm i.incom_dm i.wave if  balance_6_26==21 & wave>=19 & samp_vac2==1, robust
ologit vacci name_chi iage  femal ftch_pri univ damage i.work_dm i.incom_dm i.wave if  balance_6_26==21 & wave>=19 & samp_vac2==1, robust
/*for obtaining wave dummy results when weve19 is default*/
quiet:xi:logit vacci_dm name_chi iage  femal ftch_pri univ damage i.work_dm i.incom_dm ib19.wave if balance_6_26==21 & wave>=19 & samp_vac2==1, robust
margins, dydx(*)

/*dummy_6_10*/
/*linear name_order*/
reg vacci name_chi_6_10 iage  femal ftch_pri univ damage i.work_dm i.incom_dm i.wave if  balance_6_26==21 & wave>=19 & samp_vac2==1, robust
ologit vacci name_chi_6_10 iage  femal ftch_pri univ damage i.work_dm i.incom_dm i.wave if balance_6_26==21 & wave>=19 & samp_vac2==1, robust
/*for obtaining wave dummy results when weve19 is default*/
quiet:xi:logit vacci_dm name_chi_6_10 iage  femal ftch_pri univ damage i.work_dm i.incom_dm ib19.wave if balance_6_26==21 & wave>=19 & samp_vac2==1, robust
margins, dydx(name_chi_6_10)

/*tab3*/
/*性別交差*/

/*全体*/
reg vacci  c.name_chi##c.femal iage ftch_pri univ damage i.work_dm i.incom_dm i.wave if  balance_6_26==21 & wave>=19 & samp_vac2==1, robust
lincom  name_chi+c.name_chi#c.femal

ologit vacci c.name_chi##c.femal iage  ftch_pri univ damage i.work_dm i.incom_dm i.wave if  balance_6_26==21 & wave>=19 & samp_vac2==1, robust
lincom  name_chi+c.name_chi#c.femal

quiet xi: logit vacc_dm  name_chi_femal name_chi femal iage ftch_pri univ damage i.work_dm i.incom_dm i.wave if  balance_6_26==21 & wave>=19 & samp_vac2==1, robust
margins, dydx(name_chi_femal name_chi femal)
lincom  name_chi+ name_chi_femal
```

```
/*dummy_6_10*/
reg vacci　c.name_chi_6_10##c.femal iage ftch_pri univ damage i.work_dm i.incom_dm i.wave if
balance_6_26==21 & wave>=19 & samp_vac2==1, robust
lincom　name_chi_6_10+c.name_chi_6_10#c.femal

ologit vacci c.name_chi_6_10##c.femal iage　ftch_pri univ damage i.work_dm i.incom_dm i.wave if
balance_6_26==21 & wave>=19 & samp_vac2==1, robust
lincom　name_chi_6_10+c.name_chi_6_10#c.femal

quiet xi: logit vacc_dm　name_chi_6_10_femal name_chi_6_10 femal iage ftch_pri univ damage i.work_dm
i.incom_dm i.wave if　balance_6_26==21 & wave>=19 & samp_vac2==1, robust
margins, dydx(name_chi_6_10_femal name_chi_6_10 femal)
lincom　name_chi_6_10+ name_chi_6_10_femal


/*tab4:具体的効果は図にする*/

/*3 性別_list_交差:リストの過去の記憶ない人は含まないサブサンプル*/

/*全体：混合名簿の方が、影響が大きい*/

reg vacci　c.name_chi##c.femal##c.mix_list iage ftch_pri univ damage i.work_dm i.incom_dm i.wave if
balance_6_26==21 & wave>=19 & samp_vac2==1, robust
lincom　name_chi+c.name_chi#c.femal +c.name_chi#c.mix_list +c.name_chi#c.femal#c.mix_list
lincom　name_chi+c.name_chi#c.femal

ologit vacci　c.name_chi##c.femal##c.mix_list iage ftch_pri univ damage i.work_dm i.incom_dm i.wave if
balance_6_26==21 & wave>=19 & samp_vac2==1, robust
lincom　name_chi+c.name_chi#c.femal +c.name_chi#c.mix_list +c.name_chi#c.femal#c.mix_list
lincom　name_chi+c.name_chi#c.femal


quiet xi:logit vacc_dm　name_chi femal　mix_list name_chi_femal name_chi_mix_list femal_mix_list
name_chi_femal_mix_list iage ftch_pri univ damage i.work_dm i.incom_dm i.wave if　balance_6_26==21 &
wave>=19 & samp_vac2==1, robust
margins, dydx( name_chi femal　mix_list name_chi_femal name_chi_mix_list femal_mix_list
name_chi_femal_mix_list)
lincom　name_chi+ name_chi_femal +name_chi_mix_list+name_chi_femal_mix_list
lincom　name_chi+ name_chi_femal
```

```
/*dummy_6_10*/
reg vacci  c.name_chi_6_10##c.femal##c.mix_list iage ftch_pri univ damage i.work_dm i.incom_dm i.wave if balance_6_26==21 & wave>=19 & samp_vac2==1, robust
lincom  name_chi_6_10+c.name_chi_6_10#c.femal +c.name_chi_6_10#c.mix_list +c.name_chi_6_10#c.femal#c.mix_list
lincom  name_chi_6_10+c.name_chi_6_10#c.femal

ologit vacci  c.name_chi_6_10##c.femal##c.mix_list iage ftch_pri univ damage i.work_dm i.incom_dm i.wave if balance_6_26==21 & wave>=19 & samp_vac2==1, robust
lincom  name_chi_6_10+c.name_chi_6_10#c.femal +c.name_chi_6_10#c.mix_list +c.name_chi_6_10#c.femal#c.mix_list
lincom  name_chi_6_10+c.name_chi_6_10#c.femal


quiet xi:logit vacc_dm  name_chi_6_10 femal  mix_list name_chi_6_10_femal name_chi_6_10_mix_list femal_mix_list name_chi_6_10_femal_mix_list iage ftch_pri univ damage i.work_dm i.incom_dm i.wave if balance_6_26==21 & wave>=19 & samp_vac2==1, robust
margins, dydx( name_chi_6_10 femal  mix_list name_chi_6_10_femal name_chi_6_10_mix_list femal_mix_list name_chi_6_10_femal_mix_list)
lincom  name_chi_6_10+ name_chi_6_10_femal +name_chi_6_10_mix_list+name_chi_6_10_femal_mix_list
lincom  name_chi_6_10+ name_chi_6_10_femal



/*tab5:具体的効果は図にする*/

/*PANEL A: male & female*/

/*子供とおとなの効果：名前変化サンプルのみ＝男も少し含まれるサンプル*/

/*linear order*/
reg vacci name_chi name_adu iage  femal ftch_pri univ damage i.work_dm i.incom_dm i.wave if balance_6_26==21 & wave>=19 & samp_vac2==1 & name_adu!=name_chi , robust
ologit vacci name_chi name_adu femal iage  ftch_pri univ damage i.work_dm i.incom_dm i.wave if balance_6_26==21 & wave>=19 & samp_vac2==1 & name_adu!=name_chi , robust
quiet xi:logit vacc_dm iage name_chi name_adu femal ftch_pri univ damage i.work_dm i.incom_dm i.wave if balance_6_26==21 & wave>=19 & samp_vac2==1 & name_adu!=name_chi , robust
margins, dydx(name_chi name_adu)

/*PANEL B:  female*/
```

reg vacci iage name_chi name_adu femal ftch_pri univ damage i.work_dm i.incom_dm i.wave if balance_6_26==21 & wave>=19 & samp_vac2==1 & name_adu!=name_chi & femal==1, robust
ologit vacci iage name_chi name_adu femal ftch_pri univ damage i.work_dm i.incom_dm i.wave if balance_6_26==21 & wave>=19 & samp_vac2==1 & name_adu!=name_chi & femal==1, robust
quiet xi:logit vacc_dm iage name_chi name_adu femal ftch_pri univ damage i.work_dm i.incom_dm i.wave if balance_6_26==21 & wave>=19 & samp_vac2==1 & name_adu!=name_chi & femal==1, robust
margins, dydx(name_chi name_adu)

/*dummy_6_10: results of name_adu_6_10 cannot be available due to collinearity */
/*PANEL A: male & female*/
reg vacci name_chi_6_10 name_adu_6_10 iage femal ftch_pri univ damage i.work_dm i.incom_dm i.wave if balance_6_26==21 & wave>=19 & samp_vac2==1 & name_adu!=name_chi , robust
ologit vacci name_chi_6_10 name_adu_6_10 femal iage ftch_pri univ damage i.work_dm i.incom_dm i.wave if balance_6_26==21 & wave>=19 & samp_vac2==1 & name_adu!=name_chi , robust
quiet xi:logit vacc_dm iage name_chi_6_10 name_adu_6_10 femal ftch_pri univ damage i.work_dm i.incom_dm i.wave if balance_6_26==21 & wave>=19 & samp_vac2==1 & name_adu!=name_chi , robust
margins, dydx(name_chi_6_10 name_adu_6_10)

/*PANEL B: female*/
reg vacci iage name_chi_6_10 name_adu_6_10 femal ftch_pri univ damage i.work_dm i.incom_dm i.wave if balance_6_26==21 & wave>=19 & samp_vac2==1 & name_adu!=name_chi & femal==1, robust
ologit vacci iage name_chi_6_10 name_adu_6_10 femal ftch_pri univ damage i.work_dm i.incom_dm i.wave if balance_6_26==21 & wave>=19 & samp_vac2==1 & name_adu!=name_chi & femal==1, robust
quiet xi:logit vacc_dm iage name_chi_6_10 name_adu_6_10 femal ftch_pri univ damage i.work_dm i.incom_dm i.wave if balance_6_26==21 & wave>=19 & samp_vac2==1 & name_adu!=name_chi & femal==1, robust
margins, dydx(name_chi_6_10 name_adu_6_10)

/*table 6*/
/*PANEL A: male & female*/
reg vacci i.name_chi iage name_adu femal ftch_pri univ damage i.work_dm i.incom_dm i.wave if balance_6_26==21 & wave>=19 & samp_vac2==1 & name_adu!=name_chi ,robust
ologit vacci i.name_chi iage name_adu femal ftch_pri univ damage i.work_dm i.incom_dm i.wave if balance_6_26==21 & wave>=19 & samp_vac2==1 & name_adu!=name_chi , robust
quiet xi:logit vacc_dm i.name_chi iage name_adu femal ftch_pri univ damage i.work_dm i.incom_dm i.wave if balance_6_26==21 & wave>=19 & samp_vac2==1 & name_adu!=name_chi , robust
margins, dydx(_Iname_chi_2 _Iname_chi_3 _Iname_chi_4 _Iname_chi_5 _Iname_chi_6 _Iname_chi_7 _Iname_chi_8 _Iname_chi_9 _Iname_chi_10)

```
/*PANEL B:    female*/
reg vacci i.name_chi iage name_adu femal ftch_pri univ damage i.work_dm i.incom_dm i.wave if
balance_6_26==21 & wave>=19 & samp_vac2==1 & name_adu!=name_chi & femal==1,robust
ologit vacci i.name_chi iage    name_adu femal ftch_pri univ damage i.work_dm i.incom_dm i.wave if
balance_6_26==21 & wave>=19 & samp_vac2==1 & name_adu!=name_chi & femal==1, robust
quiet xi:logit vacc_dm i.name_chi iage name_adu femal ftch_pri univ damage i.work_dm i.incom_dm i.wave if
balance_6_26==21 & wave>=19 & samp_vac2==1 & name_adu!=name_chi & femal==1, robust
margins, dydx( _Iname_chi_2    _Iname_chi_3    _Iname_chi_4    _Iname_chi_5    _Iname_chi_6    _Iname_chi_7
_Iname_chi_8    _Iname_chi_9    _Iname_chi_10)
```

```
/*fig1a*/
graph bar data, over(id)
```
